# Identification of QTLs Associated with Oil Content in a High-Oil *Brassica napus* Cultivar and Construction of a High-Density Consensus Map for QTLs Comparison in *B. napus*

**DOI:** 10.1371/journal.pone.0080569

**Published:** 2013-12-02

**Authors:** Xiaodong Wang, Hao Wang, Yan Long, Dianrong Li, Yongtai Yin, Jianhua Tian, Li Chen, Liezhao Liu, Weiguo Zhao, Yajun Zhao, Longjiang Yu, Maoteng Li

**Affiliations:** 1 Institute of Resource Biology and Biotechnology, College of Life Science and Technology, Huazhong University of Science and Technology, Wuhan, China; 2 National Key Laboratory of Crop Genetic Improvement, Huazhong Agricultural University, Wuhan, China; 3 Hybrid Rapeseed Research Center of Shaanxi Province, Shaanxi Rapeseed Branch of National Centre for Oil Crops Genetic Improvement, Dali, China; 4 College of Agronomy and Biotechnology, Southwest University, Chongqing, China; National Key Laboratory of Crop Genetic Improvement, China

## Abstract

Increasing seed oil content is one of the most important goals in breeding of rapeseed (*B. napus* L.). To dissect the genetic basis of oil content in *B. napus*, a large and new double haploid (DH) population containing 348 lines was obtained from a cross between ‘KenC-8’ and ‘N53-2’, two varieties with >10% difference in seed oil content, and this population was named the KN DH population. A genetic linkage map consisting of 403 markers was constructed, which covered a total length of 1783.9 cM with an average marker interval of 4.4 cM. The KN DH population was phenotyped in eight natural environments and subjected to quantitative trait loci (QTL) analysis for oil content. A total of 63 identified QTLs explaining 2.64–17.88% of the phenotypic variation were identified, and these QTLs were further integrated into 24 consensus QTLs located on 11 chromosomes using meta-analysis. A high-density consensus map with 1335 marker loci was constructed by combining the KN DH map with seven other published maps based on the common markers. Of the 24 consensus QTLs in the KN DH population, 14 were new QTLs including five new QTLs in A genome and nine in C genome. The analysis revealed that a larger population with significant differences in oil content gave a higher power detecting new QTLs for oil content, and the construction of the consensus map provided a new clue for comparing the QTLs detected in different populations. These findings enriched our knowledge of QTLs for oil content and should be a potential in marker-assisted breeding of *B. napus*.

## Introduction

Rapeseed (*B. napus*, AACC, 2n = 38) is the world’s second most important oilseed crop after soybean. Rapeseed oil represents about 13.0–16.0% of the world vegetable oil production, and is also considered as a substitute for producing feedstock oils for biodiesel [1]. With the increasing demand for vegetable oil and canola-oil-based biodiesel usage, oil content has become a key factor for increasing oil production. At present a 1.0% increase in seed oil content is equivalent to an increase of 2.3–2.5% in seed yield in *B. napus* [2]. Seed oil content is a typical quantitative trait, controlled by a large number of genes and also highly influenced by environment [3], [4]. Better understanding of the genetic determinants of this trait is very important in breeding *B. napus* for oil content. QTL mapping is an effective approach to dissect the genetic mechanisms of complex quantitative traits [5], and many QTLs for oil content or fatty acid composition have been detected in various crops, such as maize [6]–[8], soybean [9]–[11], sunflower [12], [13], peanut [14], [15] and *B. juncea* [16], [17].

In *B. napus*, QTLs for seed oil content have been identified mainly by using DH populations, and 3–27 QTLs with estimated phenotypic variation in the range of 1.2–19.0% were detected in a single *B. napus* population [4], [18]–[21]. Other types of populations have also been used for *B. napus* in some cases, for example, intervarietal set of substitution lines [22] and recombinant inbred lines (RILs) [23]. However, the oil content of the parents in these populations showed only small differences, such as 48.0%×47.2% for GS (‘Sollux’×‘Gaoyou’) DH population [4], 41.8%×42.7% for MS (‘Mansholt's Hamburger Raps’×‘Samourai’) DH population [21], 43.6%×42.0% for TN (‘Tapidor’×‘Ningyou7’) DH population [20], 46.0%×48.0% for DY (‘Darmor-*bzh*’×‘Yudal’) DH population and 41.2%×46.6% for RNSL (‘Rapid’×‘NSL96/25’) DH population [19] and 40.4%×37.2% for a RIL (‘GH06’×‘P174’) population [23]. These experiments were carried out in four, three, four, three, three and seven environments [4], [18]–[21], [23] with 282, 151, 202, 442, 242, 150 and 183 lines, respectively [4], [18]–[21], [23]. More recently, there were QTL analyses for oil content using populations with larger oil content differences in their parents or more environments. Zhao *et al.* identified nine statistically significant QTLs (SL-QTLs) for seed oil content in 11 trials using the new GS DH map [24], and Sun *et al*. detected 12 QTLs in three trials using Z5 (‘zy036’×‘51070’) DH population with 92 lines whose parents had approximately 10% difference in their seed oil contents [25]. Zhao *et al.* validated and characterized that the QTL *OilA1* could influence oil content in rapeseed within the linkage group A1 using a set of near-isogenic lines (NILs) [26]. Each member of the mapping population might carry different alleles for oil content and represents its own genetic background. However, there were some limiting factors in the populations for detecting QTLs associated with oil content in previous studies: for example, populations were usually not large enough [18], [20], [21], [23], [25], trials were carried out in few environments [4], [19]–[21], [23], [25] or there were only small differences in seed oil content of the two parents [4], [18]–[21], [23], [24].

A high-density genetic linkage map is considered as a key factor for increasing statistical power and precision of detecting QTLs [27]. So far, linkage maps constructed for detecting QTLs associated with oil content in *B. napus* have been based on a range of marker systems, such as simple sequence repeats (SSRs) [18]–[20], [23]–[25], [28], sequence-related amplified polymorphisms (SRAPs) [18], [23], [24], sequence tagged sites (STSs) [20] and intron fragment length polymorphism (IFLPs) [25]. The number of marker loci in individual genetic linkage maps varied from 125 to 527, covering a total length of 1196.0–2690.0 cM with an average marker interval of 3.5–8.8 cM, respectively [18]–[21], [23]–[25], [28]. Several dense consensus genetic maps have been constructed in *B. napus* to increase marker density [29], [30]. Lombard and Delourme [31] constructed a consensus map covering a total length of 2429.0 cM by integrating three individual linkage maps. Raman *et al.* [32] constructed a consensus map, consisting of a 1359 anchored array based on diversity array technology markers, which covered a total of 1987.2 cM. Many QTLs for oil content had been identified in different populations, but the positions of these QTLs differed among various populations and the comparisons of QTLs were tenuous due to the lack of many common markers. Thus it is necessary to construct a consensus map based on individual maps for oil content and to compare the QTLs detected in different populations.

The aims of the present study were as follows: (1) to develop a large segregating DH population with parents showing >10% difference in oil content; (2) to detect QTLs for oil content through a well-constructed linkage map and the phenotypes in eight natural environments; and (3) to construct a consensus linkage map and compare the QTLs for oil content detected with those detected in other studied *B. napus* populations.

## Materials and Methods

### Plant Material

The *B. napus* segregating DH population used in this study was derived from a cross between ‘KenC-8’ and ‘N53-2’. ‘KenC-8’ was the parental of the variety ‘Zayou59’ released in 1996 in China, which was selected from the multi-way hybridized combination, ‘1721-1B’ · ‘start’×‘955’ · ‘ChunShan2B’. ‘KenC-8’ is a spring *B. napus* with seed oil content of approximately 40%. ‘N53-2’ is a DH line developed from the Canadian canola cultivar ‘Midas’ and a Chinese inbred line ‘SE8’. ‘N53-2’ is a winter-type *B. napus* with seed oil content >50%. A total of 348 DH lines were developed in the year 2007 by microspore culture applied to F_1_ plants and named the KN DH population.

### Field Trials

The field experiments were carried out in eight natural environments at four different locations. The materials were planted in a winter rapeseed area, Dali of Shannxi Province, in northwest China (coded DL) for four years (2008–2009, 2009–2010, 2010–2011 and 2011–2012); in a spring rapeseed area, Sunan of Gansu Province, in northwest China (coded GS) for two years (2010.4–2010.9 and 2011.4–2011.9); and another two semi-winter rapeseed areas, Wuhan (coded WH) and Huanggang (coded HG) of Hubei Province, in central China for one year each (2011–2012 for WH and 2010–2011 for HG). The experiment locations of WH and HG were the experiment bases of Huazhong University of Science and Technology, and DL and GS were the experiment bases of Hybrid Rapeseed Research Center of Shaanxi Province. No specific permissions were required for the field trials. Year–location combinations were treated as microenvironments, and then these microenvironments were divided into three contrasting macroenvironments: spring, semi-winter and winter. The field experiments were in a randomized complete block design with three replications in Dali and with two replications in the other sites. The DH population, together with their parents and F_1_ hybrids, were sown in double rows for each plot in all locations. There were about 12 plants per row, with a space of 0.4 m between rows and 0.2 m between plants. The field management followed normal agricultural practice. At maturity, five representative plants in the middle of each plot were bulk harvested.

### Seed Oil Content Measurement

The seed oil content in all eight trials was measured by nuclear magnetic resonance (NMR) using the method of Burns *et al*. [22] with modifications.

### Molecular Marker Assays

The genotypes of the KN DH population were analyzed using mainly two types of molecular markers: SSRs and SRAPs. In addition, several STSs markers and IFLPs primer pairs were also used to construct the genetic linkage map.

Primer sequence of SSR markers were obtained from various sources: SSR primer pairs prefixed “FITO” were developed by Iniguez-Luy *et al*. [33]; “CB” and “BRAS” were published by Piquemal *et al*. [34]; “Na”, “Ol” and “Ra” were developed by Lowe *et al*. [35]; “CNU” and “niab” were obtained from the *Brassica rapa* Genome Project (BrGP, http://www.brassica-rapa.org/); “sN”, “sR” and “sS” were provided by Agriculture and Agri-Food Canada (AAFC); “MR” were published by Uzanova and Ecke [36]; “B0”, “H0” and “S0” were developed by Ding *et al*. [37]; “BnGMS” were developed by Cheng *et al*. [38]; and “HAU” and “SA” were obtained from private communications. STS markers prefixed “IGF” were published by Qiu *et al*. [20]; “ANL2” and “CHS” by Li *et al*. (2006) [39]; ZAAS326 by Zhao *et al*. [24]; and STS primer pairs prefixed “SF” and “BrSF” as well as IFLP primer pairs GIFLP046 were developed by Sun *et al*. [25].

The sequence of SRAP markers were from the description of Li and Quiros [40]. There were 32 forward and 21 reverse primers employed (Table S1), resulting in 672 primer combinations. The forward primers were fluorescently labelled with a blue-color dye set (Applied Biosystems), namely, FAM. The SRAP products were separated with Applied Biosystems 3730xl DNA Analyzers with size standards ROX-500 (Applied Biosystems), and GeneMapper Software v3.7 (Applied Biosystems) was used to analyze the results. Each of the polymorphic loci was considered as a dominant marker [18]. The polymorphic primer pairs were named by combining the names of the forward and reverse primers, with a number to indicate the base pair of the polymorphic bands (e.g. e1m5-136).

If SSR, STS or IFLP primer pairs generated more than one polymorphic loci, small letters were used to distinguish the different loci following the marker name. For instance, two genetic loci named BRAS102a on chromosome A2 and BRAS102b on chromosome C2 were both generated from the same primer marker BRAS102.

### Linkage Map Construction

The genetic linkage map was constructed by using JoinMap software Version 4.0 [41]. Markers with a mean chi-square value ≥3.0 were excluded in all genetic groups to ensure the markers mapped to linkage groups with a fairly correct order. The threshold for ‘goodness-of-fit’ was set to ≤5.0 with a recombination frequency of <0.4 and LOD scores >1.0. Centimorgan distances were calculated by the Kosambi function for map distance [42]. Finally, all markers in the KN DH map were examined by chi-square test for ‘goodness-of-fit’ to the expected 1∶1 (P<0.01) segregation ratio.

### QTL Analysis and Meta-analysis

QTL analysis was performed by using the software Windows QTL Cartographer 2.5 (http://statgen.ncsu.edu/qtlcart/WQTLCart.htm) [43], [44]. Composite interval mapping (CIM) model was used to estimate putative QTLs with additive effect. A walking speed was set to 2 cM and a window size of 10 cM with five background cofactors. Significance levels for the LOD scores were determined by 1000-permutation test based upon a 5% experiment-wise error rate [45]. Thus, LOD of 2.94–3.10 was used to identify SL-QTLs in each environment, respectively. To avoid missing QTLs with small genetic effects, QTLs that appeared repeatedly in at least two environments at LOD <2.94–3.10 but >2.0 were considered as micro-real QTLs (MR-QTLs) [46]. The overlapping QTLs with LOD >2.0 and <2.94–3.10, as well as all SL-QTLs, were termed identified QTLs [47].

QTLs for oil content, which were detected in multi-environments located in the same region with overlapping confidence interval, may have been one single QTL [48]. Identified QTLs with overlapped confidence intervals were integrated into consensus QTLs using a meta-analysis method with BioMercator2.1 software [48], [49], which has been successfully used in *B. napus* [47], [50], [51]. The process was according to the description of Shi *et al*. [47].

The QTLs were named as described by McCouch *et al.* [52] with minor modifications. A designation begins with QTL abbreviation “*qOC*” (*q*, QTL; *OC*, oil content) suffixed with the linkage group (A1-A10, C1-C9), a hyphen (-), and finally the serial number of QTLs in the linkage group (e.g. *qOC-A2-1*).

### The Consensus Map Construction and QTL Comparison for Oil Content between Different Populations

To determine whether QTLs for oil content detected in the KN DH population were new QTLs, they were compared to QTLs from other populations in previous studies. Comparison of QTLs detected in different populations was carried out using the BioMercator2.1 software [48], [49], including a RIL population [23] and several DH populations: GS/05 [4], GS/12 [24], DY and RNSL [19], Z5 [25] and TN [20]. Although the GS/05 and GS/12 maps were constructed from the same DH population (“Sollux”×“Gaoyou”), the two maps were considered as different maps because they were constructed by different markers and QTLs were detected in different environments.

A ‘two-round’ strategy of QTL comparison was adopted [47], [50]. In the first round, QTLs identified in different populations were collected, and those QTLs in the same population with overlapped confidence intervals were integrated into consensus QTLs using QTL meta-analysis. The consensus QTLs were named with the population abbreviation followed with ‘*qOC*’, a hyphen (-) and the linkage group. If more than one consensus QTLs were found in a linkage group, a serial number of the QTL was added (e.g. *DY-qOC-A2-2*). It should be noted that those consensus QTLs in the KN DH population were named with the population abbreviation ‘*KN*’ followed with the consensus QTLs names for QTL comparison (e.g. *KN-qOC-A1-1*). In the second round, the markers in homologous chromosomes were projected from other maps on the reference map to construct a consensus map based on the common markers (sharing the same name) [48], [49]. Then the consensus QTLs detected in different populations were aligned to the consensus map.

## Results

### Seed Oil Content Analysis

**Figure 1:**
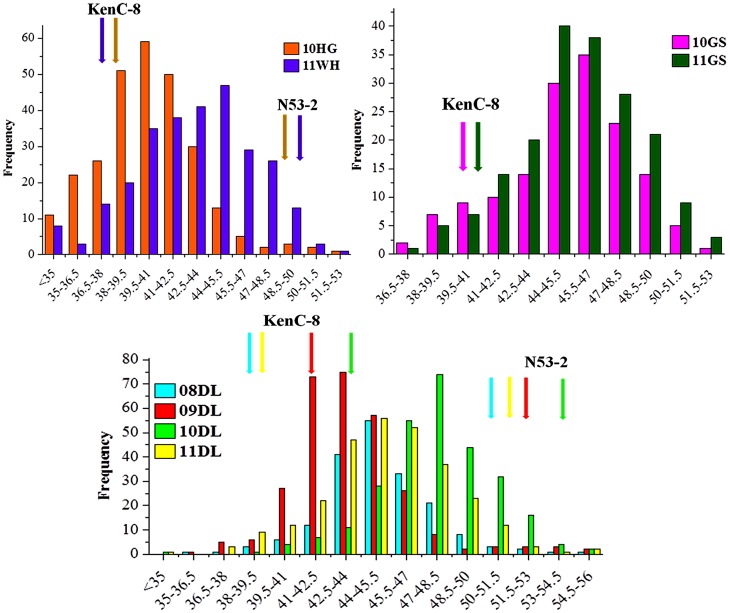
Phenotypic variation in seed oil content in the parents and KN DH population. (A), (B) and (C) indicated the frequency distribution of seed oil content in semi-winter, spring and winter macroenvironment in different years.

The frequency distributions of oil content in the KN DH population as well as the two parents in the eight microenvironments covering three macroenvironments were summarized in Fig. 1. Because of a strong requirement for vernalization, the high-oil parent ‘N53-2’ and some DH lines did not flower in the spring rapeseed area (10GS and 11GS) and these lines were treated as missing data.

There was a wide range of oil contents in all trials in this study, which showed approximately transgressive and continuous distribution as expected for characters displaying quantitative trait segregation, suggesting polygenic effects of oil content (Fig. 1). The two parents ‘N53-2’ and ‘KenC-8’ showed >10% difference in seed oil contents in six field trials, but not for trials 10GS and 11GS (Table 1). The average oil content of the KN DH population showed significant differences across trials, ranging from 42.0% in trial 10HG to 47.6% in 10DL (Table 1). In general, seed oil content in the winter macroenvironment was higher than in the semi-winter macroenvironment for both parents and DH lines (Table 1 and Fig. 1). In the same macroenvironment, oil content in different trials might also showed large differences; for example, the mean oil content for both parents and DH lines in 10DL was higher than in 09DL. Those findings suggested that the average seed oil content in *B. napus* was significantly influenced by environment.

**Table 1:** Means and ranges for seed oil content of KN DH population evaluated in eight microenvironments.

Trials^a^		08DL	09DL	10DL	10GS	10HG	11DL	11GS	11WH
KenC-8	Mean^b^	39.4±0.86	41.6±0.17	43.1±0.81	40.5±1.75	38.2±1.63	39.0±0.23	40.9±1.32	37.3±1.65
N53-2	Mean	50.7±0.61	51.7±0.58	53.8±0.12		48.6±0.75	51.4±1.28		49.5±1.44
DH	Mean	44.8±0.17	44.6±0.13	47.6±0.17	46.0±0.14	42.0±0.17	43.6±0.15	45.9±0.16	43.2±0.22
	Max	51.7	52.8	54.8	51.4	51.5	53.2	52.8	51.5
	Min	38.2	37.0	34.5	39.4	33.2	36.0	38.7	34.1

a Oil content was evaluated in eight microenvironments, where the numbers indicate the year and the letters indicate the location.

b Mean value ± SE.

### Linkage Map Construction

**Figure 2:**
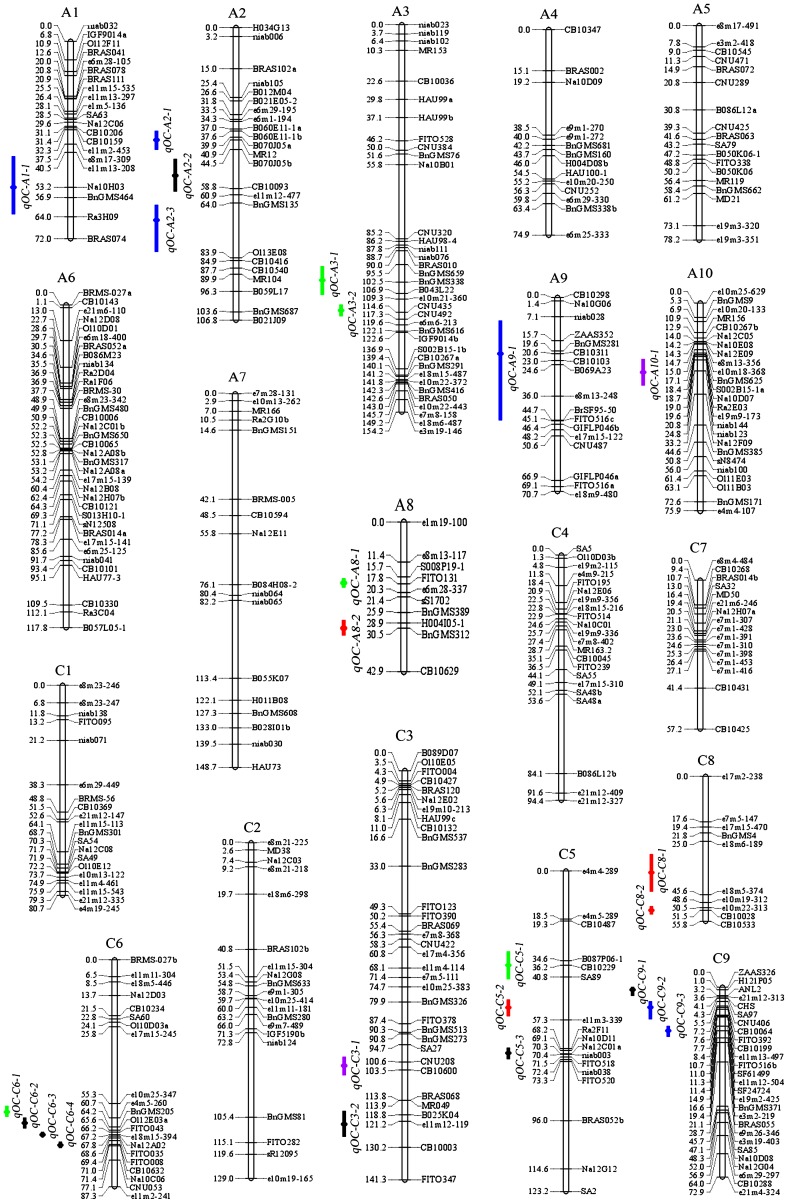
Genetic linkage map and the locations of QTLs for seed oil content in KN map. The 19 linkage groups were represented by vertical bars, designated as A1-A10 in the A genome and C1-C9 in the C genome based on multiple anchor markers located on each chromosome. The loci name were listed on the right of the linkage groups, while position of loci were showed on the left side of linkage groups, which given in Centimorgan (cM). The consensus QTLs associated with oil content in different environments were indicated by bars with various backgrounds on the left of each linkage group. (Blue bar, microenvironment specific QTLs; green bar, one macroenvironment specific QTLs; black bar, QTLs detected in two macroenvironments, red bar, QTLs detected in three macroenvironments; purple bar, MR-QTLs).

In total, 552 of 1630 molecular markers were identified as polymorphic between the two parents and were subsequently used for genetic linkage map construction using JoinMap software Version 4.0 [41]. Finally, a framework of the genetic linkage map was constructed with 403 of these markers, including 275 SSRs, 117 SRAPs, 10 STSs and 1 IFLP (Fig. 2 and Table 2). As most of the SSR and STS markers were assigned to public linkage maps, they were treated as anchor markers. The 403 markers were assigned to 19 linkage groups, named A1–A10 in the A genome and C1–C9 in the C genome according to new guidelines nomenclature for *B. napus* linkage groups (http://www.brassica.info/resource/maps/lg-assignments.php). The genetic linkage map had a total length of 1783.9 cM with an average marker interval of 4.4 cM according to the Kosambi function. Each length of the 19 linkage groups ranged from 42.9 (A8) to 154.2 cM (A3), with an average length of 93.9 cM (Fig. 2 and Table 2). The average linkage group lengths of A and C genomes were very similar, with 94.2 and 93.5 cM, respectively. In A genome, there were 217 markers in total and the number in single chromosome ranged from 10 (A8) to 36 (A3 and A6) with an average of 21.7; while 186 markers were located in C genome and the number of markers varied from 10 (C8) to 33 (C3) with an average of 20.7 in single chromosome. This suggested that A and C genomes had similar polymorphism in the KN DH population. Chromosome C9 had the highest marker density (one marker per 2.7 cM), and chromosome A7 had the lowest (one marker per 8.7 cM) (Fig. 2 and Table 2).

**Table 2:** Distribution of 403 markers on 19 linkage groups in the KN DH map.

Linkage	No. of markers				Ratio%			Length	Density	No. of markers skewed to			
Group	SRAP	SSR	STS	Total	SRAP	SSR	STS			KenC-8	N53-2	Total	Ratio%
**A1**	7	13	1	21	33.3	61.9	4.8	72.0	3.4	12	0	12	57.1
**A2**	3	20	0	23	13.0	87.0	0	106.8	4.6	0	1	1	4.3
**A3**	8	27	1	36	22.2	75.0	2.8	154.2	4.3	0	14	14	38.9
**A4**	5	9	0	14	35.7	64.3	0	74.9	5.4	0	12	12	85.7
**A5**	4	14	0	18	22.2	77.8	0	78.2	4.3	0	2	2	11.1
**A6**	6	30	0	36	16.7	83.3	0	117.8	3.3	0	8	8	22.2
**A7**	2	15	0	17	11.8	88.2	0	148.7	8.7	2	1	3	17.6
**A8**	3	7	0	10	30.0	70.0	0	42.9	4.3	4	0	4	40.0
**A9**	3	11	2	17	17.6	64.7	11.8	70.7	4.2	3	0	3	17.6
**A10**	6	19	0	25	24.0	76.0	0	75.9	3.0	0	24	24	96.0
**C1**	10	10	0	20	50.0	50.0	0	80.7	4.0	0	12	12	60.0
**C2**	9	10	1	20	45.0	50.0	5	129.0	6.5	0	17	17	85.0
**C3**	7	26	0	33	21.2	78.8	0	141.3	4.3	1	11	12	36.4
**C4**	9	13	0	22	40.9	59.1	0	94.4	4.3	0	6	6	27.3
**C5**	3	14	0	17	17.6	82.4	0	123.2	7.2	1	3	4	23.5
**C6**	7	14	0	21	33.3	66.7	0	87.3	4.2	0	14	14	66.7
**C7**	9	7	0	16	56.3	43.8	0	57.2	3.6	13	0	13	81.3
**C8**	7	3	0	10	70.0	30.0	0	55.8	5.6	8	0	8	80.0
**C9**	9	13	5	27	33.3	48.1	18.5	72.9	2.7	1	19	20	74.1
**Total**	**117**	**275**	**10**	**403**	**29.0**	**68.2**	**2.5**	**1783.9**	**4.4**	**45**	**144**	**189**	**46.9**

A large proportion (46.9%, 189/403) of the mapped loci showed segregation distortion in the KN DH map, in which 76.2% deviated toward the high-oil parent ‘N53-2’ and 23.8% toward the low-oil parent ‘KenC-8’. These distorted markers were distributed across all 19 chromosomes and tended to cluster on A3, A10, C2, C9 and C6, especially on A10 with 96.0% of its markers showing distorted segregation (Table 2). The skewed loci on linkage groups A1, C7 and C8 favored the parent ‘KenC-8’ allele, and linkage groups A3, A4, A10, C1, C2, C3, C6 and C9 comprised loci favoring the parent ‘N53-2’ allele (Table 2). In addition, no more than five skewed loci were distributed on chromosomes A2, A5, A7, A8, A9 and C5.

### SL-QTL and MR-QTL Detection and Meta-analysis for Oil Content

QTL for oil content were analyzed in each experiment by CIM approach, and identified QTLs were integrated into consensus QTLs using a meta-analysis method. Detailed information of identified QTLs was given in Table S2 and consensus QTLs were summarized in Table 3 and Fig. 2.

**Table 3:** Consensus QTLs for seed oil content in the KN DH population.

QTL	Chromosome	Position(cM)	LOD	Additive	R^2^ (%)	CI^a^	Environment^b^
*qOC-A1-1*	A1	53.2	3.99	−0.80	11.74	42.0–62.9	10GS
*qOC-A2-1*	A2	41.0	3.66	−0.60	4.42	37.6–44.5	10HG
*qOC-A2-2*	A2	53.9	3.35–5.15	−0.92–−0.60	4.74–10.26	48.1–59.6	10HG/11GS
*qOC-A2-3*	A2	70.1	4.03	−0.99	11.19	64.8–81.6	11GS
*qOC-A3-1*	A3	101.8	2.98–3.41	0.46–0.57	3.40–3.74	96.2–107.5	09DL/**11DL**
*qOC-A3-2*	A3	113.8	2.15–3.28	0.40–0.55	2.67–5.86	111.6–116.1	08DL**/09DL/11DL**
*qOC-A8-1*	A8	17.6	2.77–4.28	0.53–0.89	3.39–5.90	16.4–18.9	11WH/**10HG**
*qOC-A8-2*	A8	30.4	3.97–7.09	0.57–0.82	4.81–8.73	28.2–32.6	10HG/09DL/10DL/11DL/11GS
*qOC-A9-1*	A9	20.6	3.22	0.58	3.55	8.6–44.7	11DL
*qOC-A10-1*	A10	25.2	2.22–2.70	−0.84–−0.62	3.41–5.36	20.5–30.0	**11GS/08DL/11WH**
*qOC-C3-1*	C3	102.0	2.03–2.55	0.55–0.67	2.64–5.69	98.9–105.2	**11WH/10GS/10DL**
*qOC-C3-2*	C3	122.1	2.95–6.09	0.53–0.68	3.43–7.48	117.6–126.5	10HG/11DL/09DL/**10DL**
*qOC-C5-1*	C5	36.3	6.06–6.28	0.63–0.76	6.79–11.36	31.1–41.5	08DL/09DL
*qOC-C5-2*	C5	52.6	2.75–8.23	0.63–0.98	5.18–17.88	49.4–55.8	08DL/11WH/09DL/10HG/**11GS**
*qOC-C5-3*	C5	70.0	2.18–6.76	0.70–0.90	3.71–10.58	67.9–72.2	11DL/10GS/11DL/**11GS**
*qOC-C6-1*	C6	58.4	4.58–5.72	0.76–0.83	7.90–8.67	56.4–60.4	10DL/08DL
*qOC-C6-2*	C6	62.7	2.59–4.76	0.49–0.83	3.51–6.85	60.7–64.7	10HG**/09DL**
*qOC-C6-3*	C6	67.2	2.99–7.00	0.70–0.98	4.26–8.57	66.4–68.1	11DL/08DL/10HG/**11WH**
*qOC-C6-4*	C6	71.2	3.13–8.29	0.53–1.01	4.16–11.08	70.0–72.3	09DL/11DL/11WH/10DL
*qOC-C8-1*	C8	37.1	2.07–4.21	0.68–0.85	4.78–6.36	30.0–44.3	11DL/**11GS/11WH**
*qOC-C8-2*	C8	51.5	2.56–3.53	0.41–0.73	2.69–8.01	49.9–53.1	11DL**/10GS/09DL**/**11WH**
*qOC-C9-1*	C9	1.0	2.54–5.51	0.56–0.63	4.61–6.19	0.0–3.3	08DL/09DL/**11GS**
*qOC-C9-2*	C9	7.7	5.90	0.65	6.60	5.5–12.1	09DL
*qOC-C9-3*	C9	16.6	3.33	0.49	3.81	14.9–19.0	09DL

a The 2-LOD confidence interval of QTLs.

b The environment in which QTLs were detected. DL: Dali, a winter rapeseed area; GS: Gansu, a spring rapeseed area; WH and HG: Wuhan and Huanggang, both are semi-winter type rapeseed growing area; 08, 09, 10 and 11 denote the year of 2008, 2009, 2010 and 2011, respectively. Environment with bold indicated that the QTLs below the threshold LOD score but with LOD >2.0.

A total of 63 identified QTLs for oil content were detected in eight microenvironments, singly explaining 2.64–17.88% of the estimated phenotypic variation (Table S2). Further analysis revealed that a large proportion of the identified QTLs (42/63) were detected in C genome, with only 21 in A genome. The number of identified QTLs detected in different microenvironments also showed great differences; for example, 11 and 12 QTLs were detected in the 11DL and 09DL trials, respectively, whereas only four were detected in 10GS (Table S2).

The 63 identified QTLs were classified into two types: (i) 57 overlapping QTLs and (ii) six non-overlapping SL-QTLs. The 57 identified QTLs with overlapping confidence intervals were integrated into 18 consensus QTLs by BioMercator2.1 software (Table S2 and Fig. 2). As a result, the average confidence interval of single QTL was reduced from 13.1 to 6.3 cM. Among these consensus QTLs, two were detected in five microenvironments (*qOC-A8-2* and *qOC-C5-2*), five in four microenvironments (*qOC-C3-2*, *qOC-C5-3*, *qOC-C6-3*, *qOC-C6-4* and *qOC-C8-2*) and the remaining 11 QTLs in two to three microenvironments (Table 3 and Table S2). The genes associated with these QTLs controlling seed oil content may be more structurally important and less affected by environment. The consensus QTLs for oil content could be divided into different types based on the macroenvironments in which they were detected; for example, seven were detected in two different macroenvironments, including one expressed in both spring and semi-winter macroenvironments (*qOC-A2-2*), two were significant in both spring and winter macroenvironments (*qOC-C5-*3 and *qOC-C9-1*) and four were detected in both semi-winter and winter macroenvironments (*qOC-C3-2*, *qOC-C6-2*, *qOC-C6-3* and *qOC-C6-4*). Furthermore, six were consistently expressed in all three macroenvironments (*qOC-A8-2*, *qOC-A10-1*, *qOC-C3-1*, *qOC-C5-2*, *qOC-C8-1* and *qOC-C8-2*). Four QTLs were only repeatedly detected in the DL microenvironment in different years (*qOC-A3-1*, *qOC-A3-2*, *qOC-C5-1* and *qOC-C6-1*), which were recognized as winter macroenvironment specific QTLs; while one QTL was only repeatedly detected in 11WH/10HG (*qOC-A8-1*) and considered a semi-winter macroenvironment specific QTL. QTLs detected in multiple macroenvironments are of great importance for breeders to select materials with wide adaptability in different macroenvironments. When doing marker-assisted breeding (MAS) for special geographical region cultivars, macroenvironment specific QTLs should be paid more attention. In addition, the six non-overlapping SL-QTLs were only expressed in a specific microenvironment and were considered as microenvironment specific QTLs (Table 3 and Table S2), these QTLs were also considered to be consensus QTLs, distributed over five linkage groups (A1, A2, A3, A9 and C9). No microenvironment specific QTLs were found in 08DL, 10DL and 11WH. Two microenvironment specific QTLs (*qOC-A1-1* and *qOC-A2-3*) were strongly expressed in 10GS and 11GS, both were belonged to spring macroenvironment, and explained 11.74 and 11.36% of phenotypic variation, respectively. The two QTLs might be worthy of attention when doing MAS for spring geographical region cultivars.

The abovementioned 24 consensus QTLs were located on 11 chromosomes, including A1, A2, A3, A8, A9, A10, C3, C5, C6, C8 and C9, and each explained a mean of 3.55–11.74% of the phenotypic variation, respectively (Table S2). Two consensus QTLs (*qOC-A10-1* and *qOC-C3-1*), both with LOD value below the significance levels but repeatedly detected in three microenvironments, were regarded as MR-QTLs. The low-oil content parent ‘KenC-8’ contributed favorable alleles at five loci, which had negative additive effects and were all located on A genome, including three microenvironment-specific QTLs (*qOC-A1-1*, *qOC-A2-1* and *qOC-A2-3*), one MR-QTL (*qOC-A10-1*) and the QTL *qOC-A2-2*. The remaining 19 QTLs all had positive additive effects, indicating that they came from the high-oil parent ‘N53-2’.

### Consensus Map Construction and QTL Comparison for Oil Content between Different Populations

**Figure 3:**
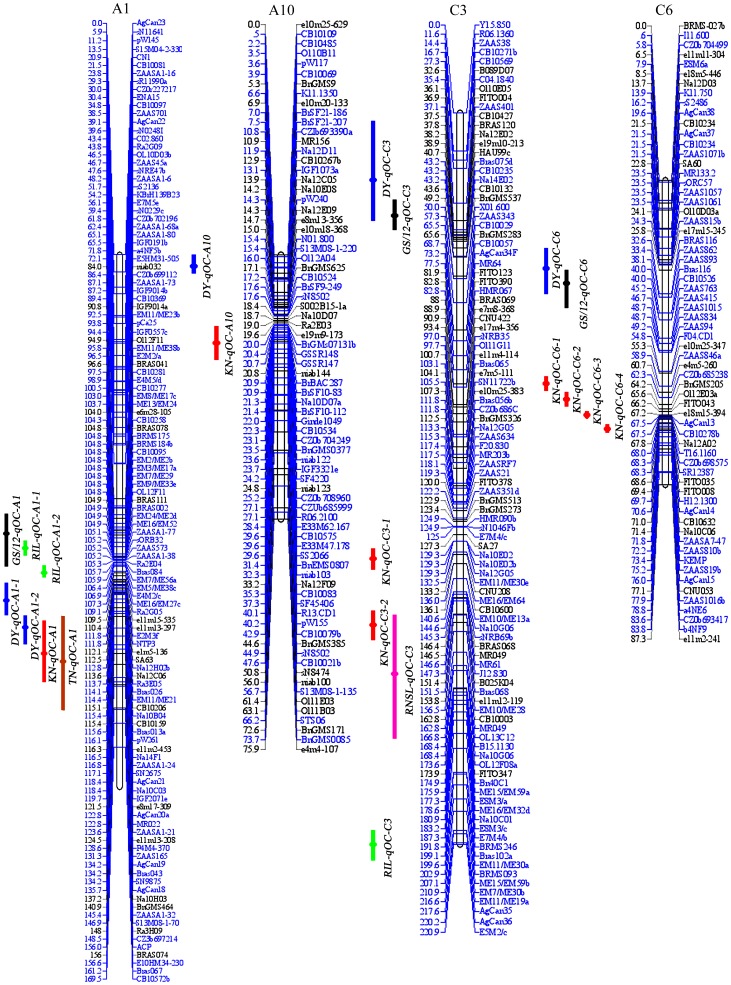
The consensus map and QTLs for oil content detected in different populations. Markers with blue color indicated these makers were projected from other maps on the KN map, *via* application of the homothetic projection based on common markers by BioMercator 2.1 software. QTLs for oil content detected in different populations were discriminated with different color bars (blue bar, QTLs detected in DY population; Orange bars, TN population; black bars, GS/12 population; green bars, RIL population; pink bars, RNSL population; red bars, KN population).

According to the reported QTLs for oil content in *B. napus*, seven populations (eight maps) were selected for comparison, including KN, DY, RNSL, TN, Z5, GS/05 and GS/12 DH population maps and a RIL population map. To exactly compare the results from different populations, QTLs detected in a single population clustered in the same region were firstly integrated to consensus QTLs by BioMercator 2.1 software. Detailed information of those consensus QTLs in different populations was summarized in Table S3. The KN map was treated as the reference map in most linkage groups, and the markers and QTLs were projected from other maps onto the KN map except for A8 and C8. On linkage groups A8 and C8, the markers and QTLs were projected from other maps onto the TN map, because few common markers were available between KN and other populations. The comparison of markers and QTLs on linkage groups A4, A9, C4 and C7 were tenuous due to the lack of common markers between different populations, and no markers and QTLs were projected from other maps onto the KN map for these linkage groups. Individual component maps were aligned to the reference linkage map to construct a consensus map. Finally, a total of 1335 markers were integrated in the consensus map. The consensus map consisted of 19 linkage groups, covering 2395.2 cM of the total genome with an average marker interval of 1.8 cM (Fig. 3 and Table S4). The length ranged from 57.2 (C7) to 220.9 cM (C3) for each individual chromosome, with an average length of 126.1 cM. Chromosome A1 had the highest marker density (one marker per 1.0 cM) and chromosome A8 had the lowest marker density (one marker per 5.9 cM) (Fig. 3 and Table S4).

A total of 99 consensus QTLs were detected in the abovementioned seven populations (eight maps) (Table S3). The number of consensus QTLs in different populations ranged from seven (TN and GS/05 populations) to 24 (KN population). Of 16 consensus QTLs, 15 were projected from the DY map onto the consensus map; however, only two of seven consensus QTLs was projected from the GS/05 map onto the consensus map. Among the 24 consensus QTLs detected in the KN DH population, 20 consensus QTLs were aligned to the consensus map. Except for the 16 consensus QTLs distributing on chromosomes A4, A9, C4 and C7 in all eight maps, 69 of the remaining 83 consensus QTLs were successfully projected from individual maps onto the consensus map (Table 4).

**Table 4:** Comparison of consensus QTLs for seed oil content detected in different populations in *B. napus*.

QTL^a^	Chr^b^	SG/12	SG/05	DY	KN	Z5	TN	RNSL	RIL
		HO×HO^c^	HO×HO	HO×MO	HO×LO	HO×LO	MO×MO	MO×LO	LO×LO
*qA1-1*	A1	*GS/12-qOC-A1-1*							*RIL-qOC-A1-1*
*qA1-2*	A1								*RIL-qOC-A1-2*
*qA1-3*	A1			*DY-qOC-A1-1*					
*qA1-4*	A1			*DY-qOC-A1-2*	***KN-qOC-A1-1***		*TN-qOC-A1*		
*qA2-1*	A2			*DY-qOC-A2-1*					
*qA2-2*	A2				***KN-qOC-A2-1***				
*qA2-3*	A2				***KN-qOC-A2-2***				
*qA2-4*	A2			*DY-qOC-A2-2*	***KN-qOC-A2-3***				
*qA2-5*	A2			*DY-qOC-A2-3*					
*qA3-1*	A3							*RNSL-qOC-A3*	*RIL-qOC-A3*
*qA3-2*	A3			*DY-qOC-A3*	***KN-qOC-A3-1***	*Z5-qOC-A3-1*			
						*Z5-qOC-A3-2*			
*qA3-3*	A3				***KN-qOC-A3-2***				
*qA5-1*	A5	*GS/12-qOC-A5-1*							
*qA5-2*	A5	*GS/12-qOC-A5-2*		*DY-qOC-A5*					
*qA6-1*	A6			*DY-qOC-A6-1*					
*qA6-2*	A6					*Z5-qOC-A6-1*		*RNSL-qOC-A6*	
						*Z5-qOC-A6-2*			
*qA6-3*	A6			*DY-qOC-A6-2*					
*qA7-1*	A7		*GS/05-qOC-A7*						
*qA7-2*	A7	*GS/12-qOC-A7*						*RNSL-qOC-A7*	*RIL-qOC-A7*
*qA8-1*	A8						*TN-qOC-A8*	*RNSL-qOC-A8*	
*qA10-1*	A10			*DY-qOC-A10*					
*qA10-2*	A10				***KN-qOC-A10-1***				
*qC1-1*	C1							*RNSL-qOC-C1*	
*qC1-2*	C1		*GS/05-qOC-C1*						
*qC2-1*	C2			*DY-qOC-C2-1*					
*qC2-2*	C2	*GS/12-qOC-C2-1*		*DY-qOC-C2-2*		*Z5-qOC-C2*	*TN-qOC-C2*		
*qC2-3*	C2	*GS/12-qOC-C2-3*							
*qC3-1*	C3	*GS/12-qOC-C3*		*DY-qOC-C3*					
*qC3-2*	C3				***KN-qOC-C3-1***				
*qC3-3*	C3				***KN-qOC-C3-2***			*RNSL-qOC-C3*	
*qC3-4*	C3								*RIL-qOC-C3*
*qC5-1*	C5				***KN-qOC-C5-1***				
*qC5-2*	C5				***KN-qOC-C5-2***				
*qC5-3*	C5				***KN-qOC-C5-3***				
*qC5-4*	C5			*DY-qOC-C5*					
*qC6-1*	C6	*GS/12-qOC-C6*		*DY-qOC-C6*					
*qC6-2*	C6				***KN-qOC-C6-1***				
*qC6-3*	C6				***KN-qOC-C6-2***				
*qC6-4*	C6				***KN-qOC-C6-3***				
*qC6-5*	C6				***KN-qOC-C6-4***				
*qC8-1*	C8	*GS/12-qOC-C8-1*							
*qC8-2*	C8							*RNSL-qOC-C8*	
*qC8-3*	C8	*GS/12-qOC-C8-2*							
*qC8-4*	C8	*GS/12-qOC-C8-3*							
*qC9-1*	C9				***KN-qOC-C9-1***				
*qC9-2*	C9				***KN-qOC-C9-2***	*Z5-qOC-C9*-1			
*qC9-3*	C9				***KN-qOC-C9-3***	*Z5-qOC-C9-2*			

a Consensus QTLs detected in different populations clustered in the same regions on the consensus map were considered to be one QTL, named a designation begins with abbreviation “*q*” suffixed with the linkage group (A1-A10, C1-C9), a hyphen (-), and finally the serial number of the QTL in the linkage group. As a result, 69 consensus QTLs aligned to the consensus map were integrated into 47 new QTLs.

b Chromosome.

c The types of population based on their parents oil content. LO: low oil content cultivar; MO middle oil content cultivar; HO: high oil content cultivar. QTLs with bold indicated that these consensus QTLs were detected in the KN DH population equivalent to the QTLs showed in Table 3.

A total of eight consensus QTLs were detected in TN, DY, RNSL, RIL, GS/05 and GS/12 populations on chromosome A1, and seven of those QTLs were aligned to the consensus map, except for one QTL in GS/05 population because of a lack of common markers (Fig. 3 and Table 4). One QTL on chromosome A1 (*KN-qOC-A1-1*) of the KN population was co-localized with QTLs *TN-qOC-A1* and *DY-qOC-A1-2*, but differed from the other four QTLs in DY, RIL and GS/12 populations. QTLs on chromosome A2 were only detected in KN, DY and Z5 populations, and one QTL *KN-qOC-A2-3* was co-localized with QTL *DY- qOC-A2-2*; the other two QTLs (*KN-qOC-A2-1* and *KN-qOC-A2-2*) were new QTLs, while the two QTLs in Z5 population were not aligned to the consensus map (Table S4). Totally, seven QTLs on chromosome A3 were successfully projected from DY, RNSL, RIL and Z5 populations onto the consensus map. One QTL on A3 (*KN-qOC-A3-1*) in KN population overlapped with QTLs Z5-*qOC-A3-1*, *Z5-qOC-A3-2* and *DY-qOC-A3*, but differed from QTLs *RIL-qOC-A3-1* and *RNSL-qOC-A3-1*; and the other QTL *KN-qOC-A3-2* was a new QTL. For the QTLs on chromosome A8, *RNSL-qOC-A8* was considered the same as QTL *TN-qOC-A8* with overlapping confidence interval. Two QTLs (*KN-qOC-A8-1* and *KN-qOC-A8-2*) were detected on chromosome A8 in the KN DH population, and *KN-qOC-A8-1* was potentially co-localized with QTLs *TN-qOC-A8* and *RNSL-qOC-A8* based on the only common marker (sS1702). *KN-qOC-A8-2* was potentially a new QTL. QTLs on chromosome A10 were only detected in KN and DY populations, and the MR-QTL *KN-qOC-A10-1* in KN population was considered a new QTL when compared with the QTL in DY population (*DY-qOC-A10*).

An interesting finding was the large number of new consensus QTLs (nine QTLs) for oil content in the C genome (Table 4), which has been rarely reported in previous studies. One QTL on chromosome C3 (*KN-qOC-C3-1*) in KN population was a new QTL compared with QTLs in DY, RNSL, RIL and GS/12 populations, and another QTL *KN-qOC-C3-2* was co-localized with QTL *RNSL-qOC-C3* (Fig. 3). Three new QTLs (*KN-qOC-C5-1*, *KN-qOC-C5-2* and *KN-qOC-C5-3*) were identified on chromosome C5 in KN population compared with QTLs in DY population. There were four QTLs (*KN-qOC-C6-1*, *KN-qOC-C6-2*, *KN-qOC-C6-3* and *KN-qOC-C6-4*) detected in KN population on chromosome C6, which differed from QTLs in DY (*DY-qOC-C6*) and GS/12 (*GS/12- qOC-C6*) populations (Fig. 3), and no QTL were found in other populations, suggesting that these were all new QTLs. QTLs on chromosome C8 were consistently detected in KN, GS/05, RNSL, Z5 and GS/12 populations, but the QTLs in KN (two QTLs), GS/05 (one QTL) and Z5 (one QTL) populations were not compared because no common markers were available. QTLs on chromosome C9 were seldom detected in previous studies except for two QTLs in Z5 population. *KN-qOC-C9-1* on chromosome C9 in KN population was a new QTL; and the other two QTLs, *KN-qOC-C9-2* and *KN-qOC-C9-3*, were co-localized with QTLs *Z5-qOC-C9-1* and *Z5-qOC-C9-2* in Z5 population, respectively (Table 4 and Table S4). Compared with the abovementioned previous studies, 14 of the 24 consensus QTLs in KN population were considered as potential new QTLs in the present study. So far, QTLs for oil content have been observed in all 19 linkage groups, but the QTLs were not well distributed between linkage groups, and the different types of populations showed different capacities for detecting QTLs. Some QTLs were repeatedly detected in different populations and formed QTL clusters in some chromosome regions, while some QTLs were only detected in specific populations. Those QTLs repeatedly detected in different populations offer the possibility of fine mapping and map-based cloning of genes contributing to seed oil content in *B. napus.* This method provided a new clue for comparing QTLs detected in different populations.

## Discussion

Seed oil content is an important trait for *B. napus*, controlled by complex genetic architecture and influenced by environment. High-oil cultivars may contain excellent alleles for high oil content and using high-oil rapeseed cultivars to construct populations may give higher resolution for QTL screening [25]. Many DH populations have been constructed for QTL analysis of seed oil content in *B. napus* [4], [19]–[25], with the differences in seed oil content between parents in the range of 0.8–5.9% and population sizes of 150–442 lines; except for the Z5 population with approximately 10% difference in seed oil content between parent but with only 92 DH lines. A DH population (KN DH) was constructed in the present study, in which the parents showed significant differences in oil content, with 11.3, 10.1, 10.7, 10.4, 12.4 and 12.2% differences in 08DL, 09DL, 10DL, 10HG, 11DL and 11WH trials, respectively. In addition to an excellent performance in oil content, the KN DH population consisted of 348 lines and to our knowledge is the largest *B. napus* DH population with >10% difference in oil content of parents.

### The New Constructed Larger DH Population and Linkage Map were Suitable for Detecting QTLs Associated with Oil Content

A DH genetic linkage map covering a total length of 1783.9 cM with an average marker interval of 4.4 cM was constructed using the KN DH population. The marker order in the KN linkage map was in good agreement with an international TN DH reference map for *B. napus* (http://brassica.nbi.ac.uk/) that has been widely used for mapping different agronomic traits [20], [46], [47], [50], [51]. A high percentage of markers (46.9%) in the KN DH map showed segregation distortion, as also observed in other *B. napus* DH populations, such as Z5 [25], MS [21], RNSL [19], DY [19] and GS/12 [24] populations with 35.9, 11.2, 20, 35.7 and 48% of markers with segregation distortion, respectively. Segregation distortion has been identified as a strong evolutionary force [53]. The unequal segregation of alleles results from a variety of different mechanisms, such as segregation distortion regions on chromosomes [54], [55], genetic hitchhiking effect [56], [57], strong zygotic selection, certation and gamete selection [58]. Interestingly, the skewed loci on chromosome A1 favored the low-oil parent ‘KenC-8’ alleles and the low-oil QTL (*qOC-A1-1*) with negative additive effect was also located on the region of skewed loci clustering. The skewed loci on chromosomes A10, C6 and C9 favored the high-oil parent ‘N53-2’ alleles and QTLs on these chromosomes were all high-oil QTLs with positive additive effect distributed over the region of skewed loci clusters. Previously, Zhao *et al.* [4] identified 35.2% of the mapped makers with significant deviations and two QTLs for oil content located on the region of skewed loci clusters in GS/05 DH population. Zhao *et al.* [24] identified 48.0% of mapped makers with distorted segregation in the GS/12 DH population and three QTLs situated in the genomic regions with distorted segregation of the marker loci. These findings suggested that segregation distortion was partially associated with QTLs for oil content in DH populations constructed from high-oil *B. napus* cultivars; however, the relationships with other types of populations require further research.

A large population, a high-density genetic map and replicated experiments in multiple environments are considered as three key factors for increasing statistical power and precision in detecting QTLs [27]. Consensus QTLs for oil content detected in previous studies were 7, 16, 11, 7, 10, 12 and 12 in TN, DY, RNSL, GS/05, RIL, Z5, GS/12 populations, respectively, and the DY population showed the most consensus QTLs (16 QTLs) with the largest population size of 442 lines (Table S3). Comparison of QTLs between the Z5 and KN populations supported the view that a larger mapping population enabled higher capacity to identify QTLs and correctly estimate the magnitude of their effects. Both of the populations showed approximately 10% difference in their parents’ seed oil contents, but the KN (348 lines) was about four times larger than the Z5 population (92 lines), as a result, the number of consensus QTLs detected in the KN population (24 QTLs) was twice that in the Z5 population (12 QTLs). Sun *et al.* [25] reported that the proportion of phenotypic variation explained by an individual QTL in Z5 population was higher than in most other reported populations, which were in the range of 9.15–24.56%. As the Z5 population was smaller than most other populations, the phenotypic variation explained by an individual QTL might be overestimated, whereas that in KN population was in the range of 2.64–17.88%. This phenomenon was also found in other studies, for example, Bradshaw *et al.* [59] reported that double the number of QTLs for 12 floral traits was detected when the F_2_ sample size increased from 93 to 465 plants, and the lowest phenotypic variation explained by an individual QTL decreased from 18.7 to 3.3% in monkey flower (*Mimulus*). In a simulation study, Li *et al.* [60] pointed out that as the population size increased from 100 to 500, the estimated QTL position and the effect asymptotically approached their true values. These results indicated the importance of population size in QTL mapping, and the use of large populations could distinguish a higher level of allelic variation and improve mapping efficiency.

Mapping populations constructed from parents with great differences in seed oil content could enable the identification of QTL associated with large phenotypic differences in seed oil content in *B. napus* [25]. The parents in most studied populations showed only small differences (usually <5%) in seed oil content except for Z5 [19]–[24]. The QTLs obtained in GS/05 and GS/12 (282 lines) population were compared with QTLs detected in KN population, as the GS population had the most similar population size to the KN population but the two populations were significantly different in their parents’ seed oil contents. The results revealed that the consensus QTLs detected in KN population (24 QTLs) were three and two times that detected in the GS/05 (seven QTLs) and GS/12 (12 QTLs) populations, respectively (Table S3). In addition, although the DY (442 lines) was larger than the KN population (348 lines), there were less QTLs detected in DY population (16 QTLs) associated with seed oil content because two parents used in DY population only showed approximately 2% difference in oil content. The results indicated that populations with significant differences in their parents’ seed oil contents might have more different alleles resulting in increasing oil content and could give a higher power of detection for QTLs. Exploring the genetic diversity in varieties’ genetic backgrounds such as in the KN population would be helpful to identify more effective alleles for increasing oil content.

### QTLs Comparison and New QTLs Detection for Oil Content in the KN Population

Generally speaking, consensus maps can greatly increase map resolution, and might be a powerful tool to survey the genetic diversity of loci/alleles underlying complex traits, develop molecular breeding and map-based cloning of genes. So far, consensus maps have been constructed for many plants, such as barley [61]–[64], bread wheat [65], sorghum [66], sunflower [67], cowpea [68] and rye [69]. However, few consensus maps have been constructed in *B. napus*. Lombard and Delourme and Raman *et al.* [31], [32] constructed consensus maps consisting of 540 and 1359 marker loci from the integration of three and six DH mapping populations, respectively, and covered a total of 2429.0 and 1987.2 cM. In order to compare the difference in QTLs for oil content detected in the KN with other populations, a consensus genetic map was constructed based on the common marker loci from different populations. Lombard and Delourme [31] predicted that the length of the *B. napus* genome was between 2127 and 2480 cM. By combining the eight individual maps into a consensus map, the length of the KN map increased from 1783.9 to 2395.2 cM with the average marker interval reduced from 4.4 to 1.8 cM, which indicated that this consensus map might have a near-complete coverage of the *B. napus* genome. This newly constructed consensus map comprised different genetic backgrounds for the eight individual maps derived from the 14 parents involved, and could be of nearly universal use as a reference map for QTL mapping in *B. napus*. In addition, most of the linkage gaps of >15 cM in the KN map were filled by the addition of 932 molecular markers to the reference map, with the exception of gaps on chromosomes A4, A9, C4, C7 and C8 (Table S4). Compared with consensus genetic maps reported for *B. napus*, the present consensus map has more markers or greater total length and more complicated genetic backgrounds. This high-density consensus map should facilitate the selection of polymorphic markers in important chromosomal intervals and provides a framework for comparing QTLs associated with oil content detected in different populations.

According to oil content of the parents used to construct the *B. napus* population for QTL analysis associated with oil content, the parents could be factitiously divided into three types: low-oil (LO, oil content <42.0%), middle-oil (MO, oil content 42.0–47.0%) and high-oil (HO, oil content >47.0%) parents. Thus the segregating populations in the present study were divided into six types based on parents’ oil contents: HO×HO (SG/05, SG/12), HO×MO (DY), HO×LO (Z5), MO×MO (TN), MO×LO (RNSL) and LO×LO (RIL) populations. Projecting the QTLs from different population maps on the consensus map showed that 14 of 24 QTLs identified in the KN DH population were new QTLs, including five new QTLs in A genome and nine in C genome. All of the six types of populations had QTLs on chromosomes A1 and C3, two QTLs detected in the KN population on C3 were new QTLs, and one QTL on A1 was co-localized with QTLs in TN and DY populations (Table 4). QTLs on chromosomes A3 and A8 were detected in four of the population types (HO×MO, HO×LO, MO×LO and LO×LO), and one of two QTLs on A3 (*KN-qOC-A3-2*) detected in the KN population was a new QTL and one of two QTLs on A8 (*KN-qOC-A8-2*) was a potential new QTL.

Zhao *et al.* [24] showed that QTLs for oil content on A1 and C3 could be detected in all six maps used in their comparison. Delourme *et al.* [19] reported that QTLs for oil content on A1 and A3 were detected in five and six populations, respectively. The results revealed that QTLs for oil content on these chromosomes appeared to be more consistent and could be detected in various gene pools. The reason might be in part that those QTLs affecting oil content were more structurally important and less affected by environment. However, QTLs on some chromosomes might only be specific to one or more particular genetic backgrounds. QTLs on chromosome C6 were detected in HO×HO (GS/12), HO×MO (DY) and HO×LO (KN); and QTLs on chromosomes A2, A10 and C5 were only detected in the HO×MO (DY) and HO×LO (KN and/or Z5) populations, which had one parent that was a special high-oil cultivar (Table 4), indicating that high-oil rapeseed cultivars may contain excellent alleles for oil content on those chromosomes. Our QTLs on A2, A10, C5 and C6 were all new QTLs (10 QTLs) except for one QTL on chromosome A2 (*KN-qOC-A2-3*) that co-localized with QTL in DY population (*DY-qOC-A2-2*). Sun *et al.* [25] reported that they had first mapped two QTLs on chromosome C9 in Z5 population. One of our QTLs (*KN-qOC-C9-1*) on chromosome C9 was a new QTL and the other two QTLs were co-localized with QTLs detected in Z5 population. Since the QTLs on chromosome C9 were only detected in KN and Z5 populations, and both were HO×LO population and had a high-oil parent with seed oil content of approximately 50%, it appeared that the alleles increasing seed oil content on chromosome C9 were only expressed in cultivars with particularly high oil contents. These findings revealed a complex genetic determinism underlying seed oil content among various *B. napus* cultivars. Many other quantitative traits have also showed that some QTLs could be detected in various gene pools, while others might only be specific to particular genetic backgrounds. For example, QTLs for erucic acid on A8 were detected in most populations, but QTLs on A6, C2 and C8 chromosomes were only detected in special individual populations [20], [21], [70]. QTLs for seed yield on A3, A10 and C4 were detected in different populations [18], [71], [72], and QTLs consistently associated with flowering time across populations were repeatedly identified on A2, A3 and C7 [46], [71], [73]. As every segregating population has its own potentiality to reveal genetic limiting factors, it will be possible to combine the alleles increasing seed oil content from different genetic background in MAS.

## Supporting Information

Table S1 The sequence of thirty-two forward and twenty-one reverse primers of SRAP markers.
(XLS)

Table S2 Identified QTLs and consensus QTLs detected in eight experiments for seed oil content in the KN population.
(XLS)

Table S3 Detailed information of QTLs and the consensus QTLs identified in eight populations.
(XLS)

Table S4 The consensus map and the location of consensus QTLs detected in different populations.
(DOCX)
