# Supplementary material for: Identification of QTLs Associated with Oil Content in a High-Oil Brassica napus Cultivar and Construction of a High-Density Consensus Map for QTLs Comparison in B. napus
Source: PLoS One. 2013 Dec 2;8(12):e80569. doi: 10.1371/journal.pone.0080569 (PMC3846612; doi:10.1371/journal.pone.0080569)
Supplement: Table S4 — The consensus map and the location of consensus QTLs detected in different populations. (DOCX) [file pone.0080569.s004.docx]

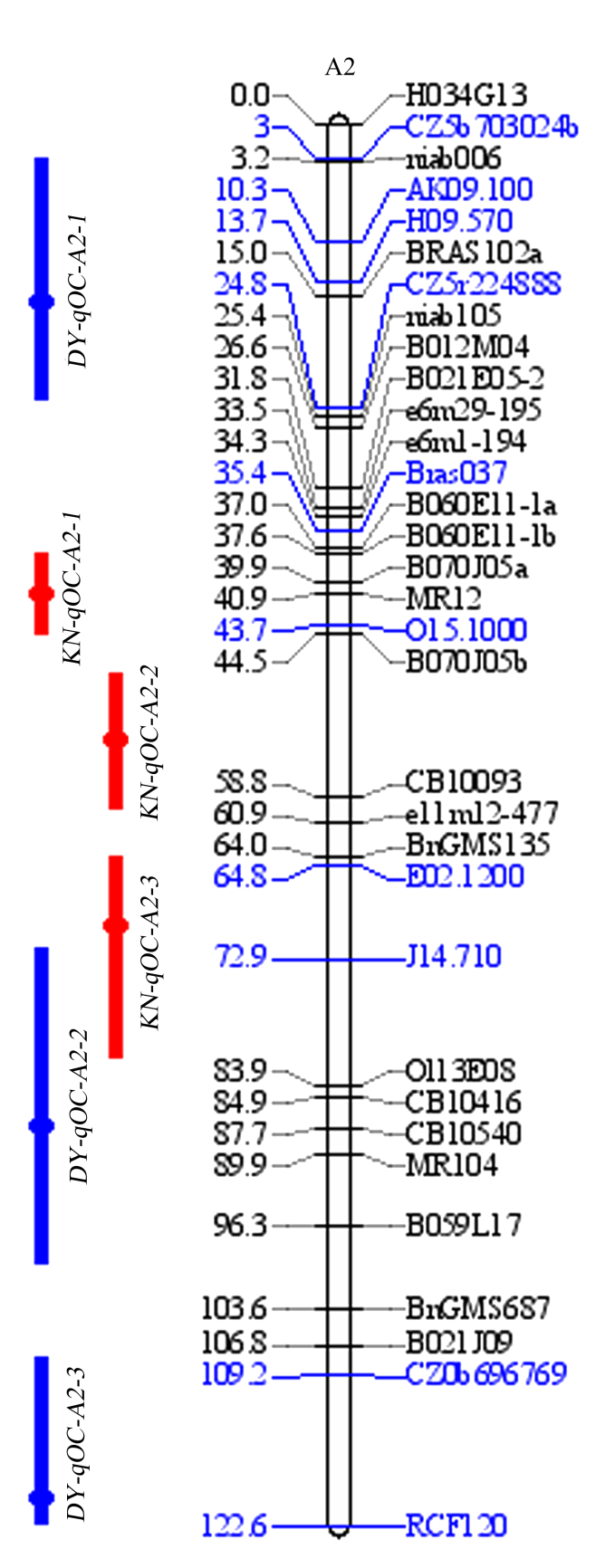

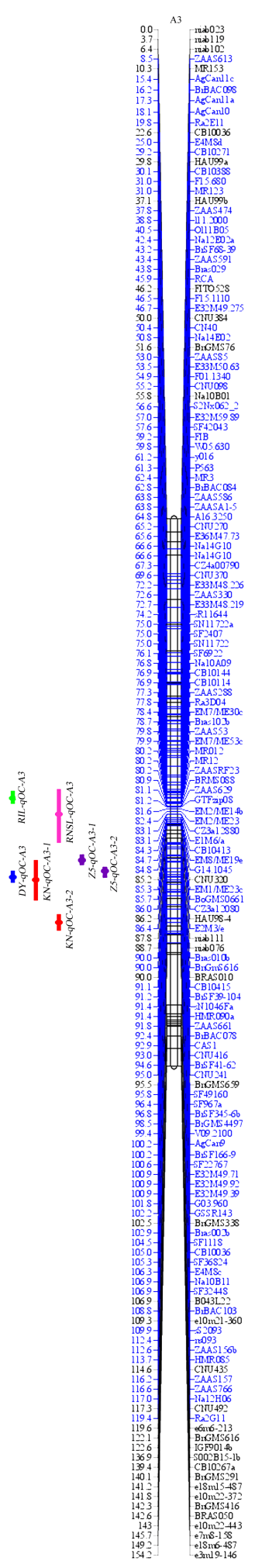

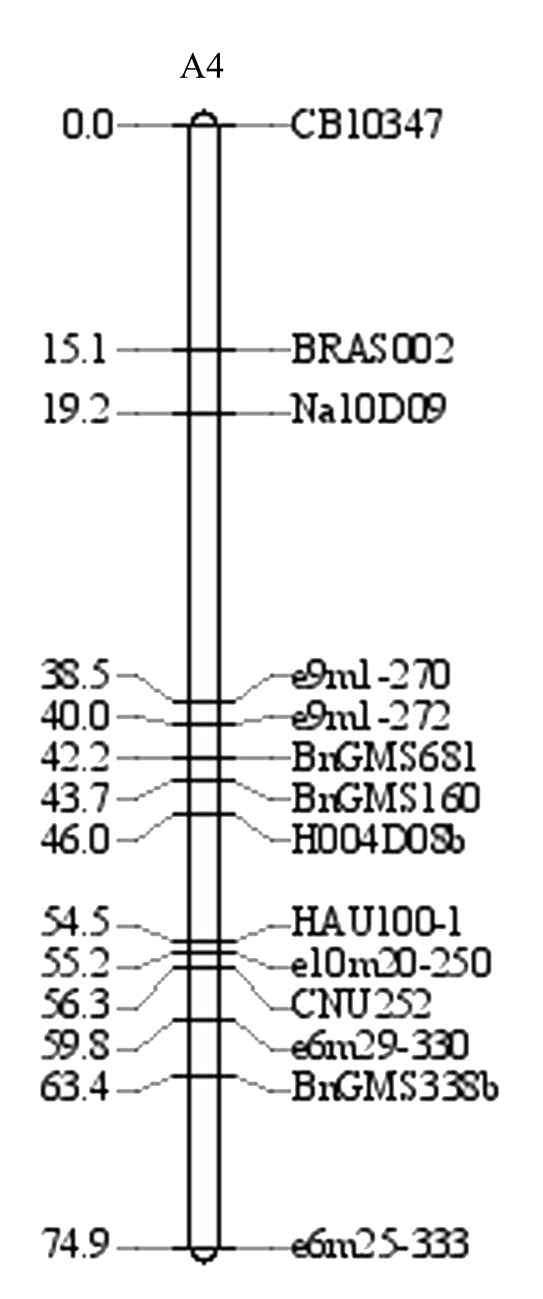

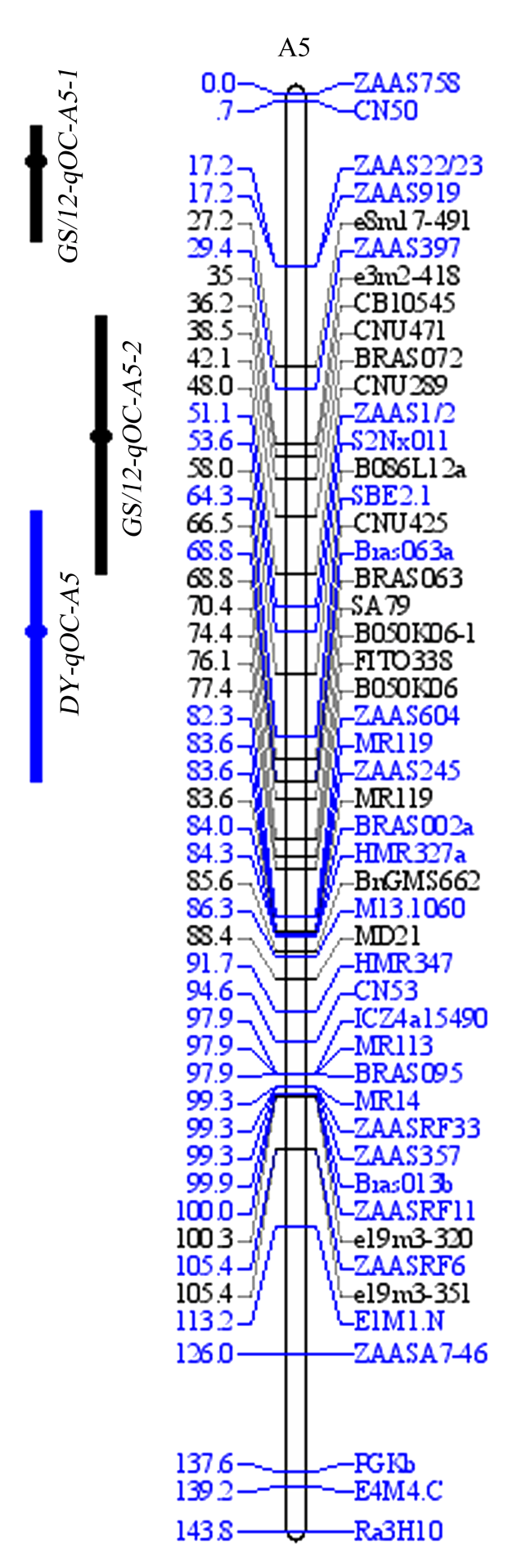

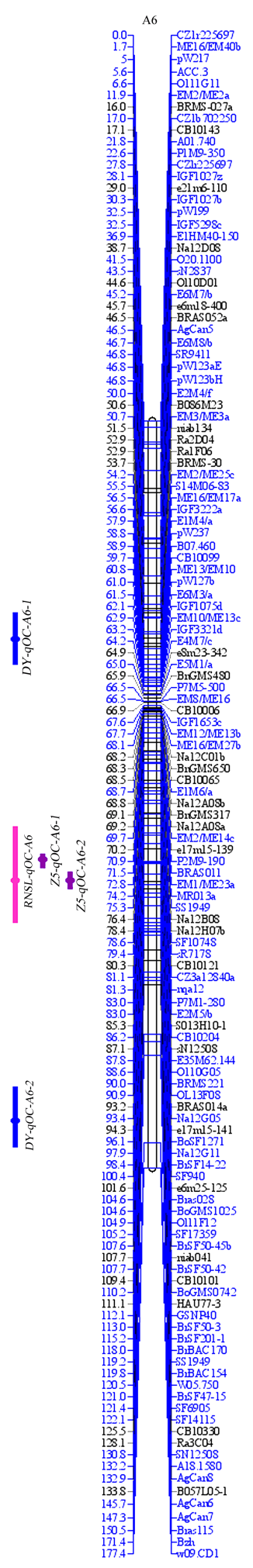

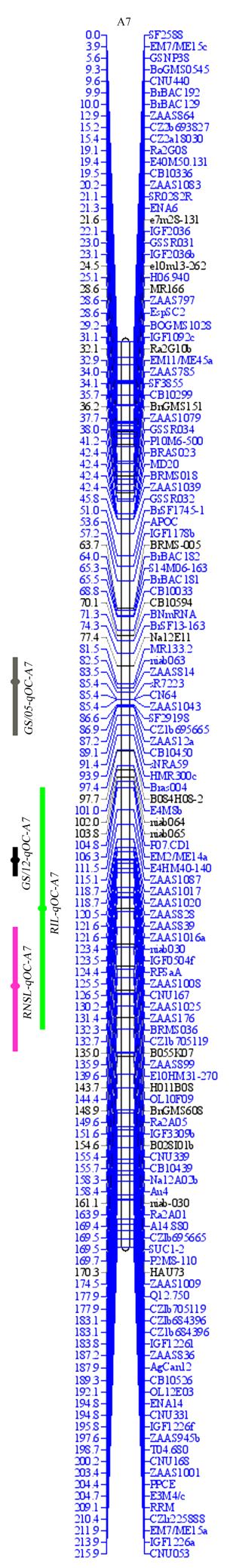

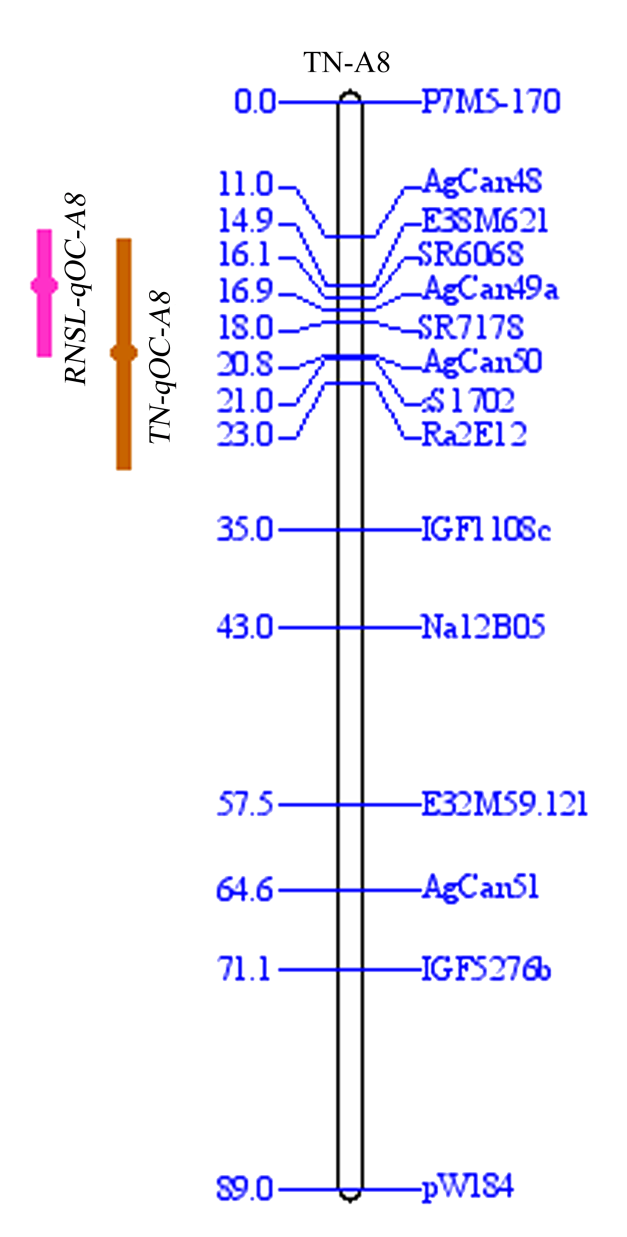

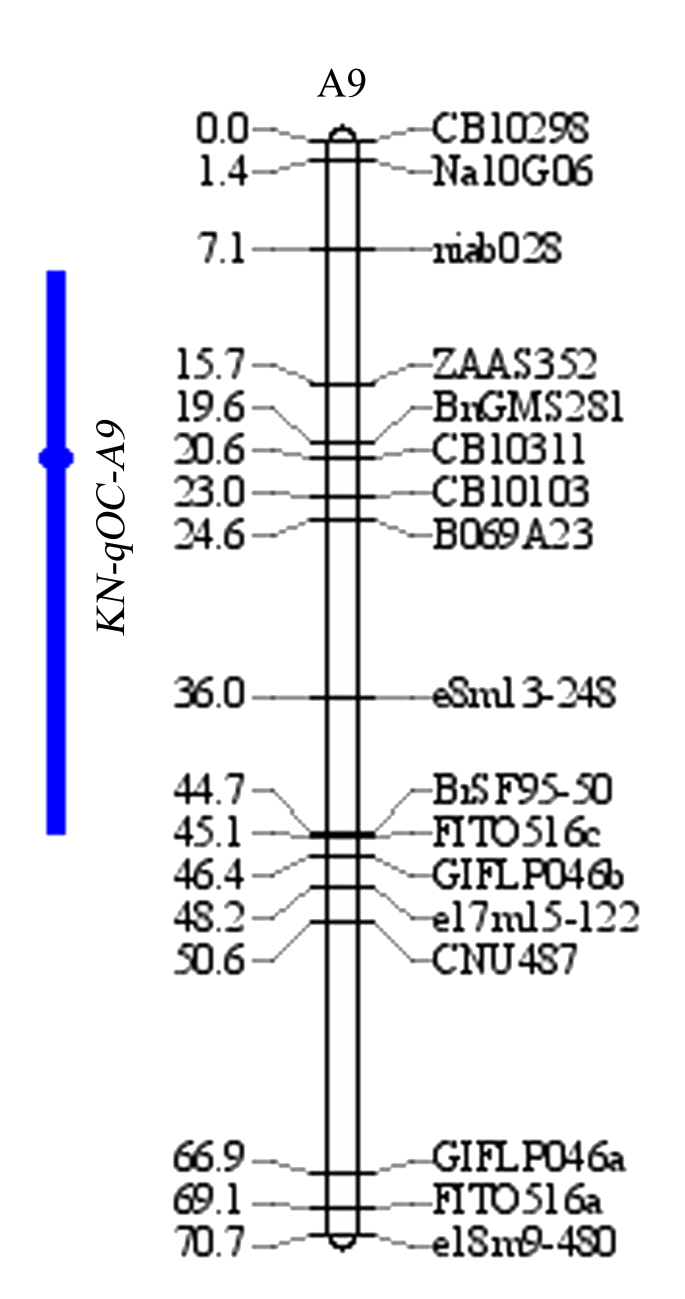


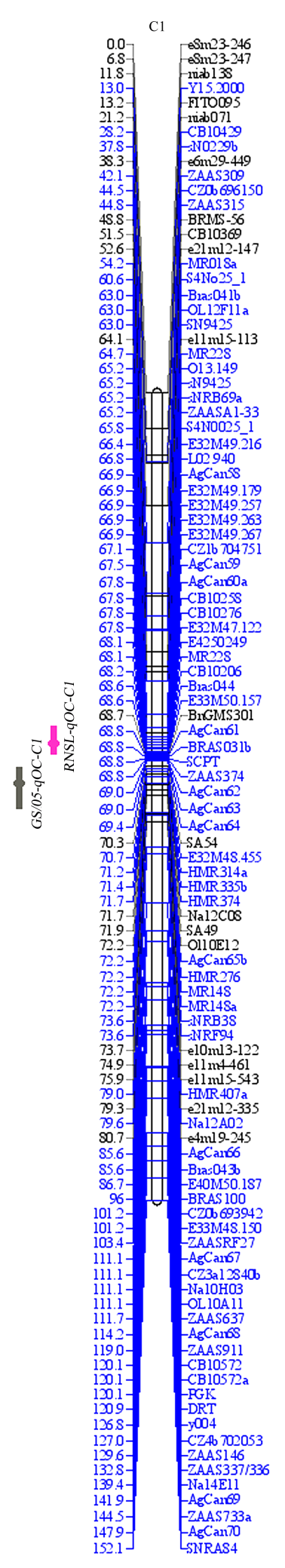

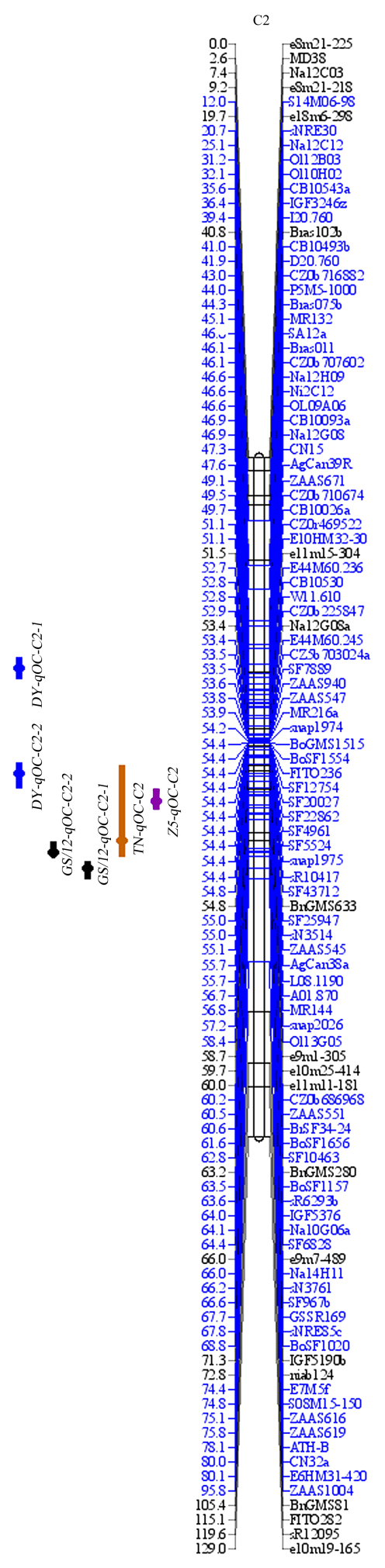


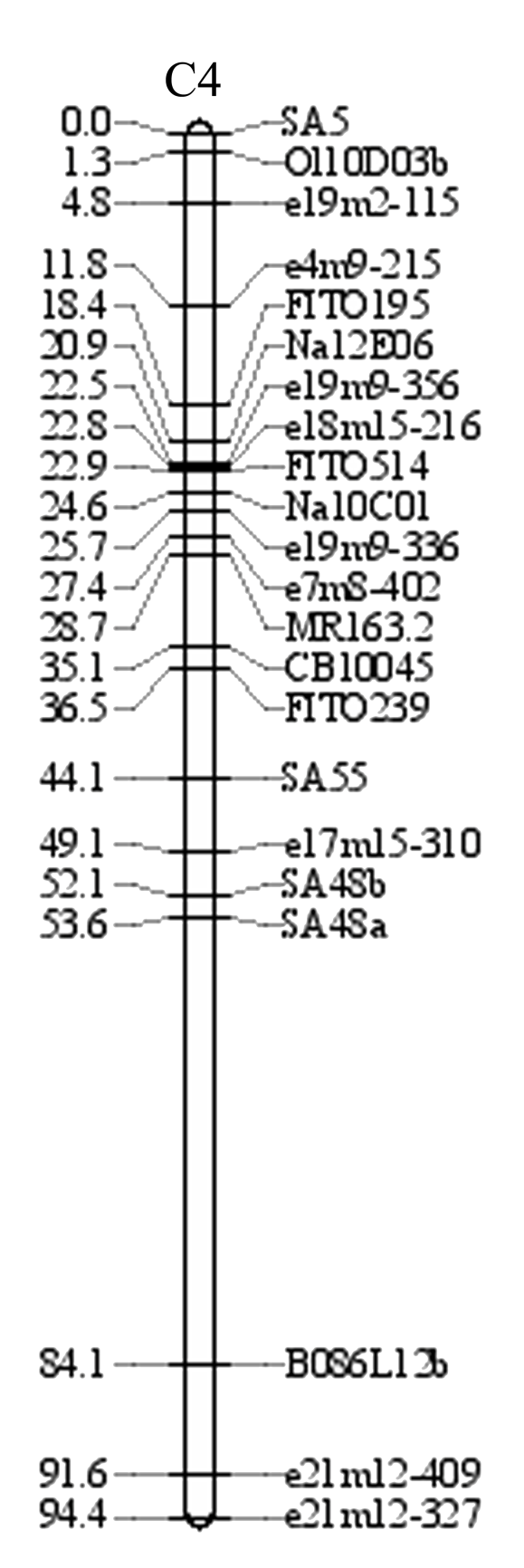

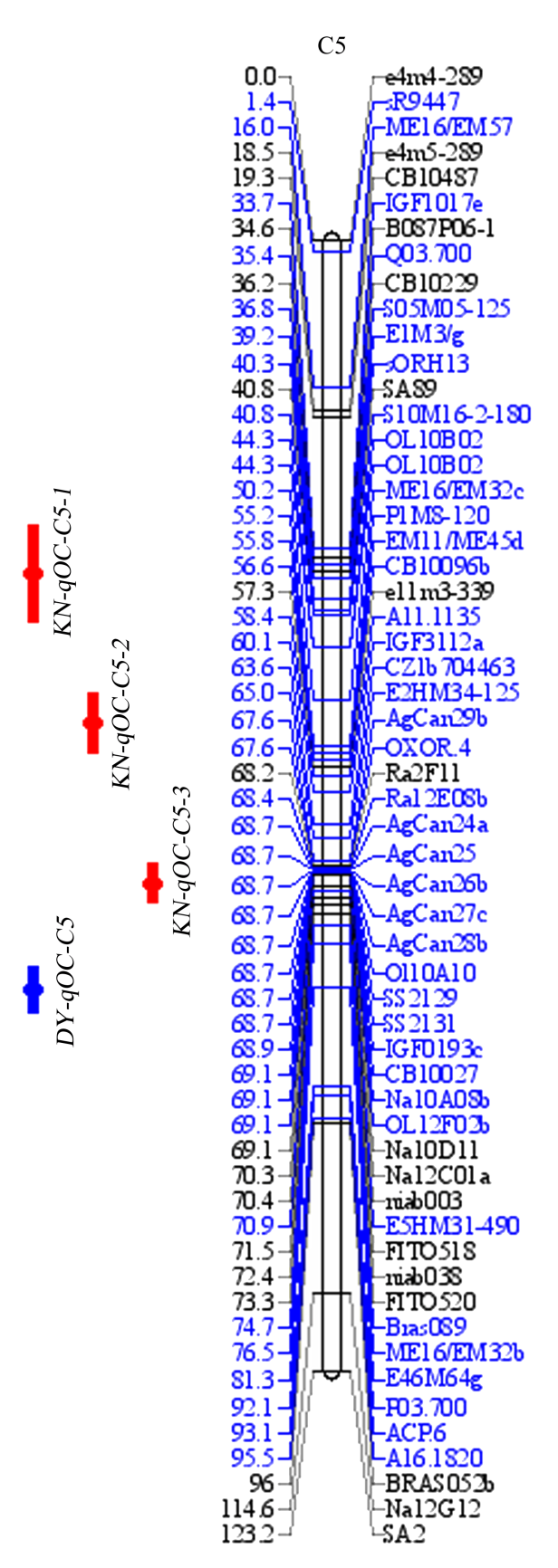


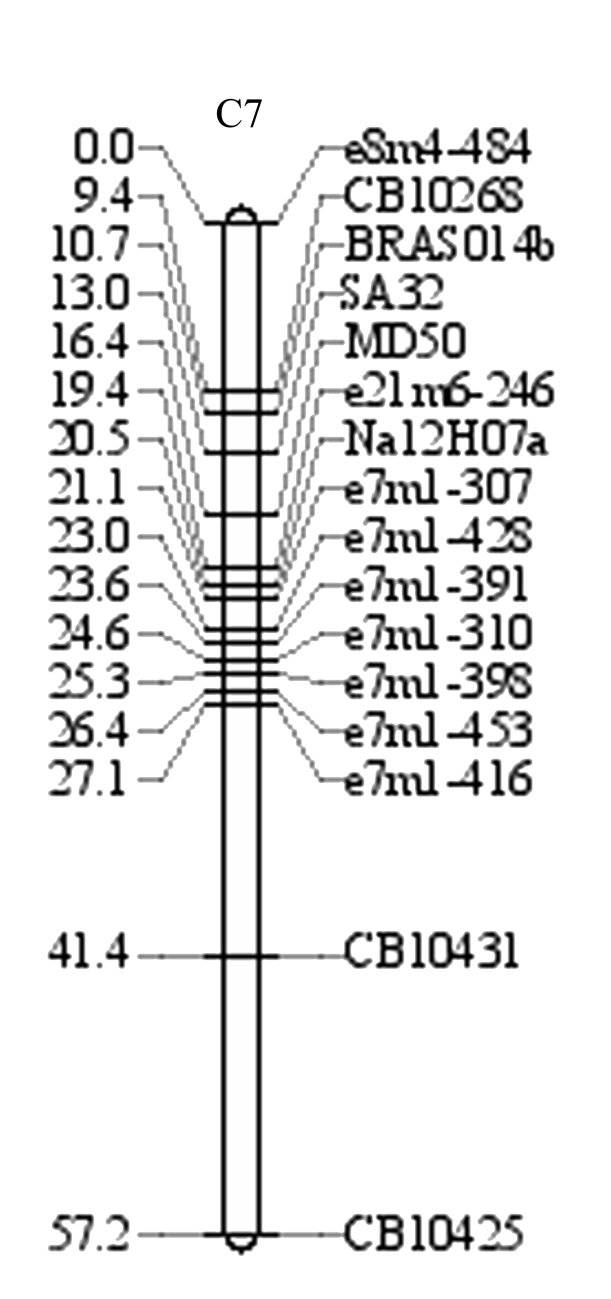

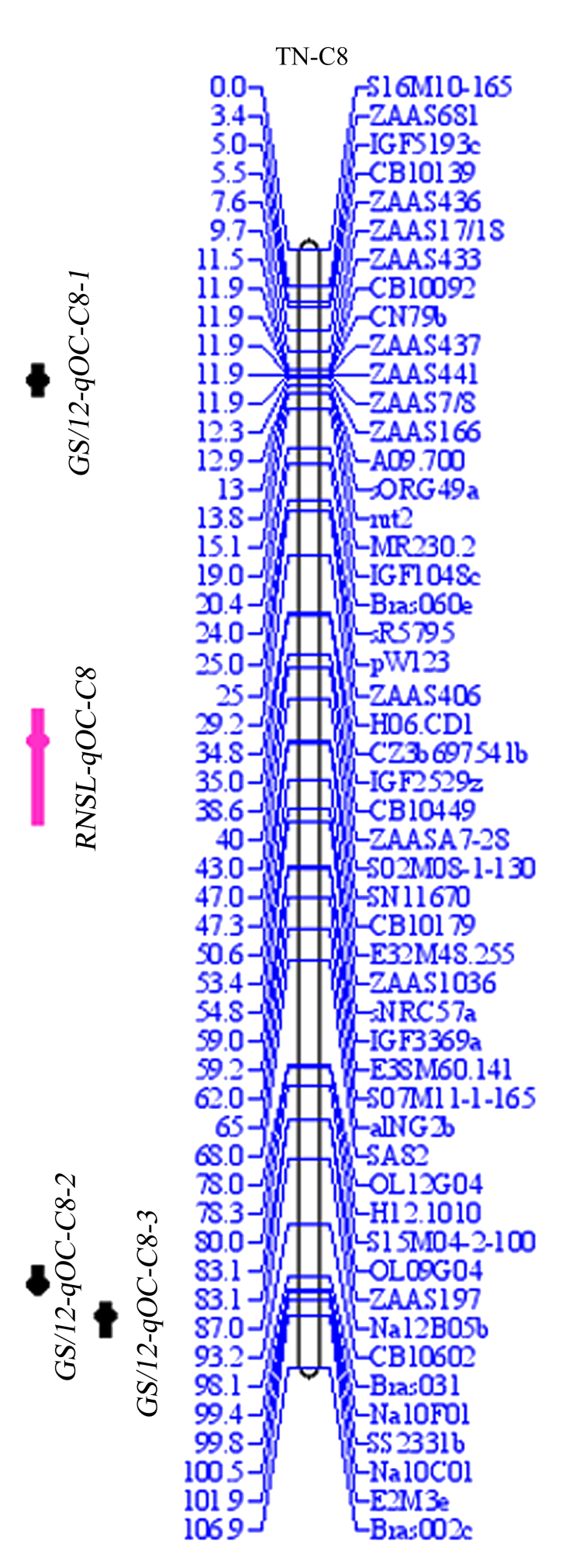

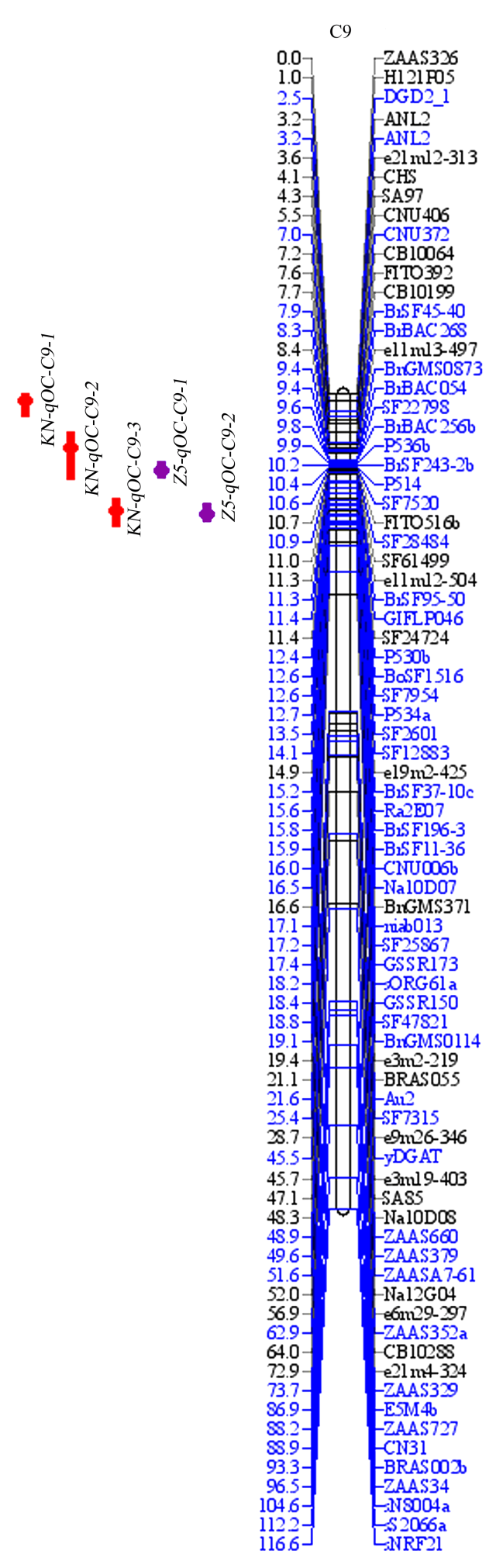


Markers with blue color indicated those makers projected from other maps on KN map, *via* application of the homothetic projection based on common markers by BioMercator 2.1 software. QTL detected in different populations were discriminated with different color bars (purple bars, QTL detected in Z5 population; blue bar, DY population; Orange bars, TN population; black bars, GS/12 population; gray bars, GS/05 population; green bars, RIL population; pink bars, RNSL population; red bars, KN population).
